# Virtual Screening of TADF Emitters for Single-Layer OLEDs

**DOI:** 10.3389/fchem.2021.800027

**Published:** 2021-12-16

**Authors:** Kun-Han Lin, Gert-Jan A. H. Wetzelaer, Paul W. M. Blom, Denis Andrienko

**Affiliations:** Max Planck Institute for Polymer Research, Mainz, Germany

**Keywords:** TADF, computer screening, OLED, chemical design, single-layer

## Abstract

Thermally-activated delayed fluorescence (TADF) is a concept which helps to harvest triplet excitations, boosting the efficiency of an organic light-emitting diode. TADF can be observed in molecules with spatially separated donor and acceptor groups with a reduced triplet-singlet energy level splitting. TADF materials with balanced electron and hole transport are attractive for realizing efficient single-layer organic light emitting diodes, greatly simplifying their manufacturing and improving their stability. Our goal here is to computationally screen such materials and provide a comprehensive database of compounds with a range of emission wavelengths, ionization energies, and electron affinities.

## Introduction

For obtaining efficient organic light-emitting diodes (OLEDs), it is convenient to tune individual processes, such as charge injection, balanced hole and electron transport, and triplet and singlet exciton harvesting, by using dedicated layers. Every new material adds a degree of freedom and hence flexibility to the OLED design. For instance, doped charge transport layers ensure Ohmic injection, an appropriate host material balances transport inside the emitting layer, and the phosphorescent emitter ensures triplet harvesting. However, every new emitter requires optimization of the surrounding layers, with respect to energy levels, triplet energies, and charge-transport properties, complicating the OLED design.

Recently, it was demonstrated that a complex multilayer design can be substituted by a simple single-layer architecture (Kotadiya et al., 2019a) without compromising the balanced and trap-free electron and hole transport. The ohmic charge injection and the absence of heterojunctions resulted in extremely low operating voltages and thus power efficiency in a single-layer OLED utilizing thermally activated delayed fluorescence, which helps to convert triplet into singlet excitons (Uoyama et al., 2012; Godumala et al., 2019)^.^ An external quantum efficiency of 19% was achieved. Owing to the broad recombination zone and low operating voltages, one of the key features of the single-layer device is the improved device stability, which can be used to design a stable blue OLED, a grand challenge in OLED research (Heimel et al., 2018; Paterson et al., 2019, 2020). In view of this, it would be useful to understand if the single-layer design can be employed for blue OLEDs: the issue here is the trap-free transport for both holes and electrons, which sets limits on the transport gap. In this paper, we first formulate the chemical design rules for TADF emitters with ambipolar transport. Using these rules, we then computationally pre-screen a set of molecules comprised of acceptor, donor, and bridge blocks and grade them according to the predicted emission wavelength.

## Design Criteria

### Singlet-Triplet Energy Splitting

**FIGURE 1:**
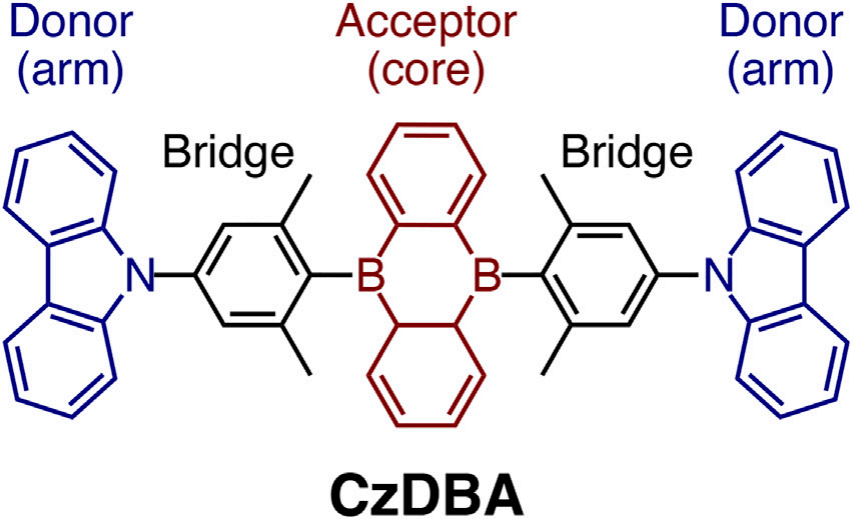
The molecular structure of a prototypical single-layer TADF emitter, 5,10-bis(4-(9H-carbazol-9-yl)-2,6-dimethylphenyl)-5,10-dihydroboran-threne (CzDBA). It features a D- π -A- π -D (or arm-bridge-core-bridge-arm) molecular architecture.

The important task of a TADF emitter is to convert triplet into singlet excitations. To do this, the reverse intersystem crossing rate, $k_{\text{rISC}}$ , should be high, which is only possible if the energy difference between the first singlet and the first triplet excited state is small, $\text{Δ}E_{\text{ST}}<0.1\;\text{eV}$ . A typical example of a TADF emitter is CzDBA (Wu et al., 2018), shown in Figure 1. CzDBA has a D- π -A- π -D architecture: two carbazole (Cz) fragments, two *m*-xylene bridges and a central 5,10-dihydroboranthrene (DBA) core. The methyl groups on the *m*-xylene bridge ensure that the core unit is nearly orthogonal to the π bridge, leading to a small overlap between the highest occupied molecular orbital (HOMO) and the lowest unoccupied molecular orbital (LUMO) and hence nearly zero $\text{Δ}E_{\text{ST}}$ .

### Ambipolar Trap-free Transport

To ensure a broad recombination zone within the emission layer, the thin film of the TADF emitter should provide balanced and trap-free transport of holes and electrons. To realize this, one needs to select compounds with an ionization energy (IE) and electron affinity (EA) lying within the trap-free energy window (Kotadiya et al., 2019b), i.e., with ionization energy (IE) < 6.5 eV and electron affinity (EA) > 2.5 eV. These criteria ensure that contaminants such as oxygen or water do not serve as energetic traps for holes and electrons.

### Small Energetic Disorder

From a dipolar glass model, the energetic disorder present in a disordered molecular solid is proportional to the dipole moment of the composing molecule. Therefore, thin organic films with molecules with a small dipole moment (*D*) normally have a narrower density of states (Novikov and Vannikov, 2009; Lin et al., 2019; Mondal et al., 2021; Stankevych et al., 2021). This design criteria can be enforced by selecting centrosymmetric molecules only of the D- π -A- π -D or A- π -D- π -A type, similar to CzDBA. This molecular architecture ensures a small dipole moment and hence narrow density of states (Liu et al., 2021).

## Building Blocks

**FIGURE 2:**
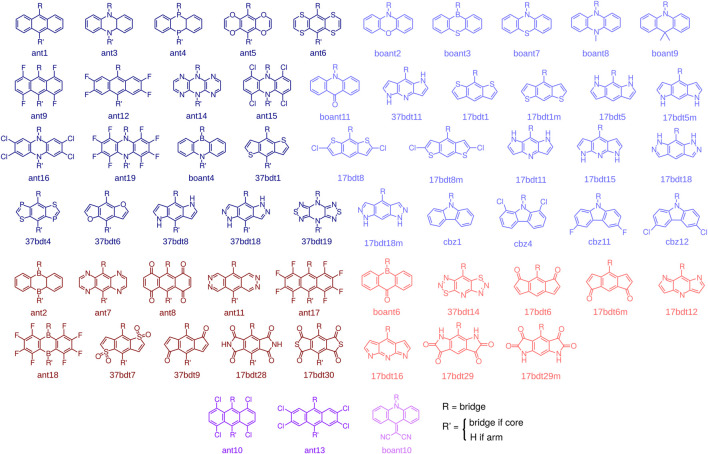
Donors (blue), acceptors (red), and building blocks that can serve both as donors and acceptors (purple). The blocks with inversion symmetry (dark colors) can be used as either core or arm fragments. The building blocks without inversion symmetry (light colors) can only be used as arm fragments. We also included boant4, which is not centrosymmetric, as a core fragment to increase the number of compounds in the database.

With these design rules in mind, and in view of the successful example of CzDBA, we build and characterize a database of emitters that fulfill the aforementioned criteria. To construct the emitters, we start with 97 potential donor and acceptor building blocks, all shown in the Supplementary Note S1. All of them are (quasi-)linear, composed of three (fused) rings and are reported in literature (synthesizable). These building blocks are further pre-screened to ensure the desired donor-acceptor architecture in an emitter. The pre-screening proceeds as follows: knowing that the IE and EA of CzDBA is already quite close to the boundary of the trap-free window (Kotadiya et al., 2019a; Liu et al., 2021) we take the IE_Cz_ and EA_DBA_ as the pre-screening criteria for donors and acceptors, respectively. Only the fragments possessing IE < IE_Cz_ + 0.2 eV (EA > EA_DBA_ - 0.2 eV) will be chosen as “trap-free” donors (acceptors) and enter the next round, see Supplementary Figure S2. The molecular structures of donors and acceptors that pass the prescreening step are summarized in Figure 2 *.* To build the emitter molecules, only the building blocks with the inversion symmetry are used as *core* fragments. These are shown in dark colors in Figure 2. This choice helps to fulfill the centrosymmetric requirement for the entire molecule.

## Computational Workflow

Using the selected building blocks, we constructed the database of D- π -A- π -D and A- π -D- π -A. The simplified molecular-input-line-entry system (SMILES) strings of compounds were created through combination of the SMILES strings of the composing donor, bridge and acceptor. The initial geometry of each compound was first optimized using a semi-empirical method and then by density functional theory (DFT). Details are given in the Supplementary Note S2.

To obtain reliable predictions of solid-state IE, EA and excited-state energy, we followed the cost-effective ω -tuning protocol (Sun et al., 2016, 2017). In addition to the $\text{Δ}E_{\text{ST}}$ , the difference in the characters of the singlet and triplet excited states are crucial to the rISC rate (El-Sayed, 1963). For this reason, the excited-state characters were evaluated using a fragment-based method (Plasser, 2020).

**FIGURE 3:**
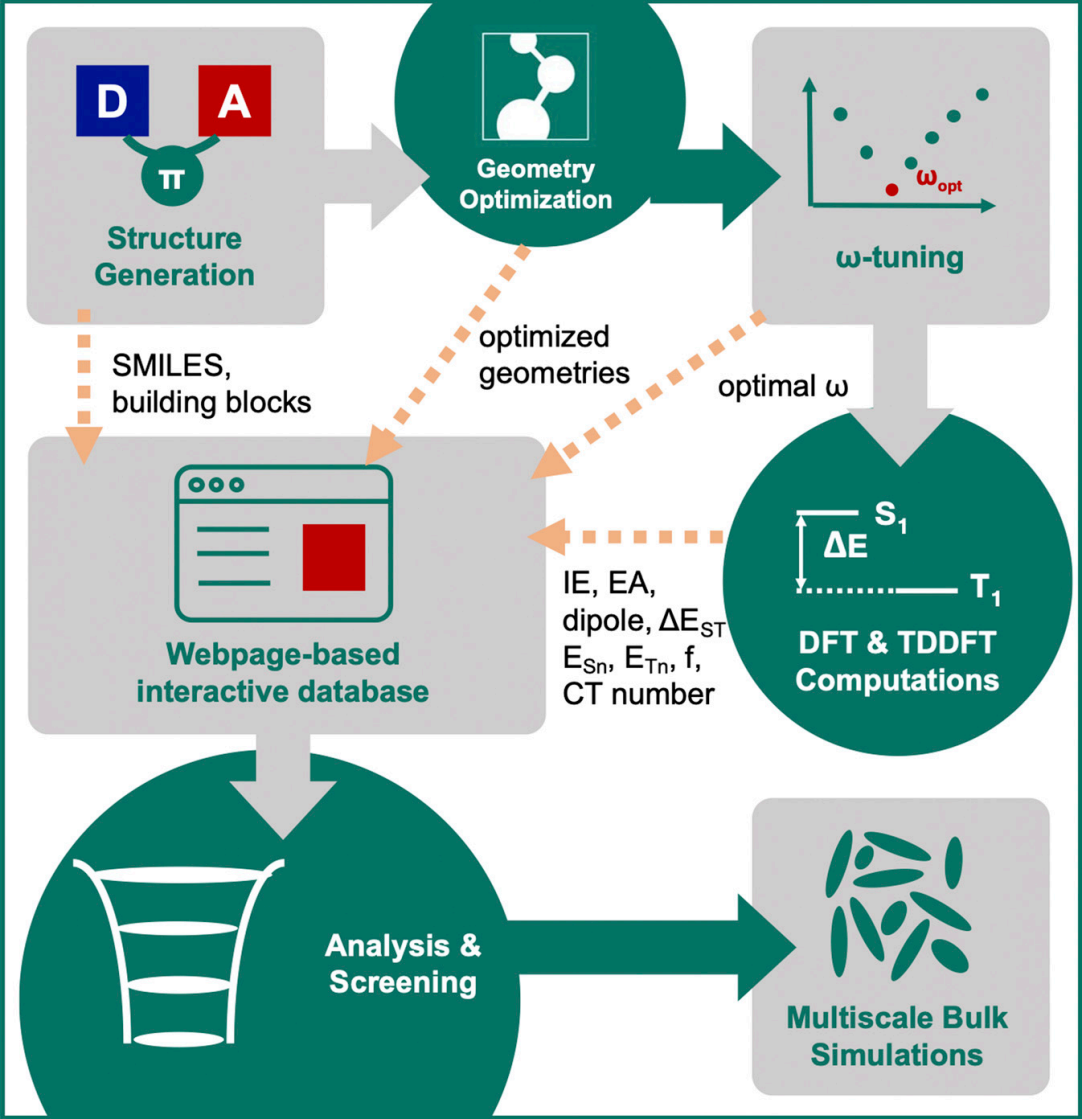
Illustration of the computational workflow for virtual screening of single-layer TADF emitters.

For compounds that pass the screening criteria, the density-of-states distributions for holes and electrons were computed via multi-scale simulations, that include morphology generation using molecular-dynamics simulations, followed by polarizable force-field evaluation of the solid-state contributions to the gas-phase energy levels (Rühle et al., 2011; Poelking and Andrienko, 2016; Andrienko, 2018; Mondal et al., 2021). The entire workflow is illustrated in Figure 3.

## Results and Discussion

### Compounds With Small Singlet-Triplet Splitting

The combination of the core and the arm fragments gives in total 441 A- π -D- π -A and 504 D- π -A- π -D compounds. Due to convergence problems in geometry optimization, especially in the anionic state with implicit solvent, the final database contained 433 A- π -D- π -A and 481 D- π -A- π -D compounds.

**FIGURE 4:**
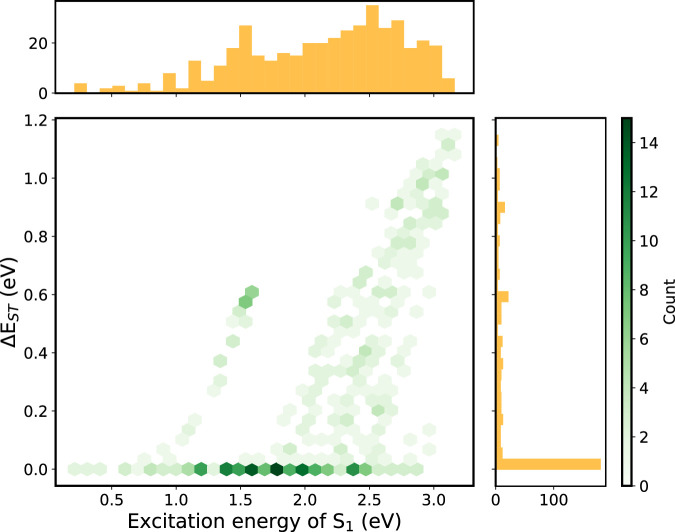
2D histogram constructed using the descriptors ( $E_{S_{1}}$ , $\Delta E_{ST}$ ) of the A- π -D- π -A database (433 molecules). The corresponding 1D histogram for each descriptor is shown on the axes.

The IE and EA of all compounds either lies within the “trap-free window” or close to the borderline of the window, showing that the effectiveness of prescreening of the building blocks. Therefore, we put our emphasis on the small $\text{Δ}E_{\text{ST}}$ criterion. The distributions of the E_S1_ and ΔE_ST_ are shown in Figure 4 (A- π -D- π -A) and Supplementary Figure S2 (D- π -A- π -D). Around 50% of the compounds (206 out of 433 for A- π -D- π -A and 268 out of 481 for D- π -A- π -D) have very small singlet-triplet energy level splitting, $\text{Δ}E_{\text{ST}}$ < 0.1 eV, which illustrates the efficiency of the design strategy, that is the use of the *m*-xylene bridge. Moreover, the computed $S_{1}$ energy and $\text{Δ}E_{\text{ST}}$ of CzDBA is 2.487 and 0.016 eV, which is in excellent agreement with the experimental values of 2.48 and 0.033 eV (Wu et al., 2018; Kotadiya et al., 2019a).

Among these small- $\text{Δ}E_{\text{ST}}$ compounds, we observed a broad distribution in the $S_{1}$ energy, ranging from 0.2 to 2.9 eV. This indicates the opportunity to design single-layer emitting OLEDs of different colors, including the infrared region. The two branches in Figure 4 represent the rest (50%) of the emitters with $\text{Δ}E_{\text{ST}}$ > 0.1 eV, where a similar branch is also observed for D- π -A- π -D (Supplementary Figure S3). This is counterintuitive as the $\text{Δ}E_{\text{ST}}$ should be small if the HOMO and the LUMO are separated *vi*a the *m*-xylene bridges.

### Analysis of the Excited-State Character

To better understand the origin of the large $\text{Δ}E_{\text{ST}}$ , we calculated the charge transfer (CT) number ranging from 0 to 1, using the fragment-based analysis (see Supplementary Note S2). We define the core as one fragment (f_C_) and two bridge + arm pairs as the other fragment (f_A_). If the hole is 100% located at one fragment and the electron is 100% located at the other one, the charge transfer number is 1, representing a 100% CT character. In contrast, if the hole and the electron are both localized on the same fragment, the CT number is 0, featuring a local-excitation (LE) character. In most cases, the CT number is a fraction between 0 and 1 since most adiabatic excited states exhibit a mixture of CT and LE characters. The larger the CT number of the excited state is, the higher the CT character it has.

**FIGURE 5:**
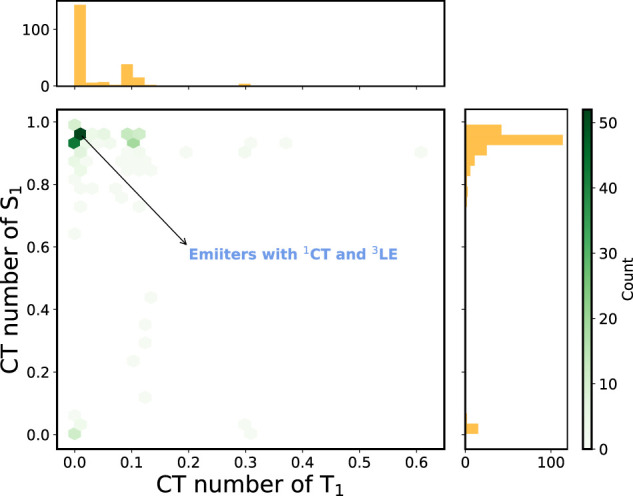
2D histogram constructed using the CT numbers of T_1_ and S_1_ states of the A- π -D- π -A molecules with $\Delta E_{ST}$ > 0.1 eV (227 molecules). The corresponding 1D histogram for each descriptor is shown on the axes.

Figure 5 depicts the 2D histogram based on the CT numbers of T_1_ and S_1_ states for the 227 A- π -D- π -A compounds with $\text{Δ}E_{\text{ST}}$ > 0.1 eV. Most of the scatter points are located at the upper left corner, meaning that these emitters possess a charge-transfer S_1_ and locally-excited T_1_ states. A similar result was observed in the D- π -A- π -D case (see Supplementary Figure S4).

**FIGURE 6:**
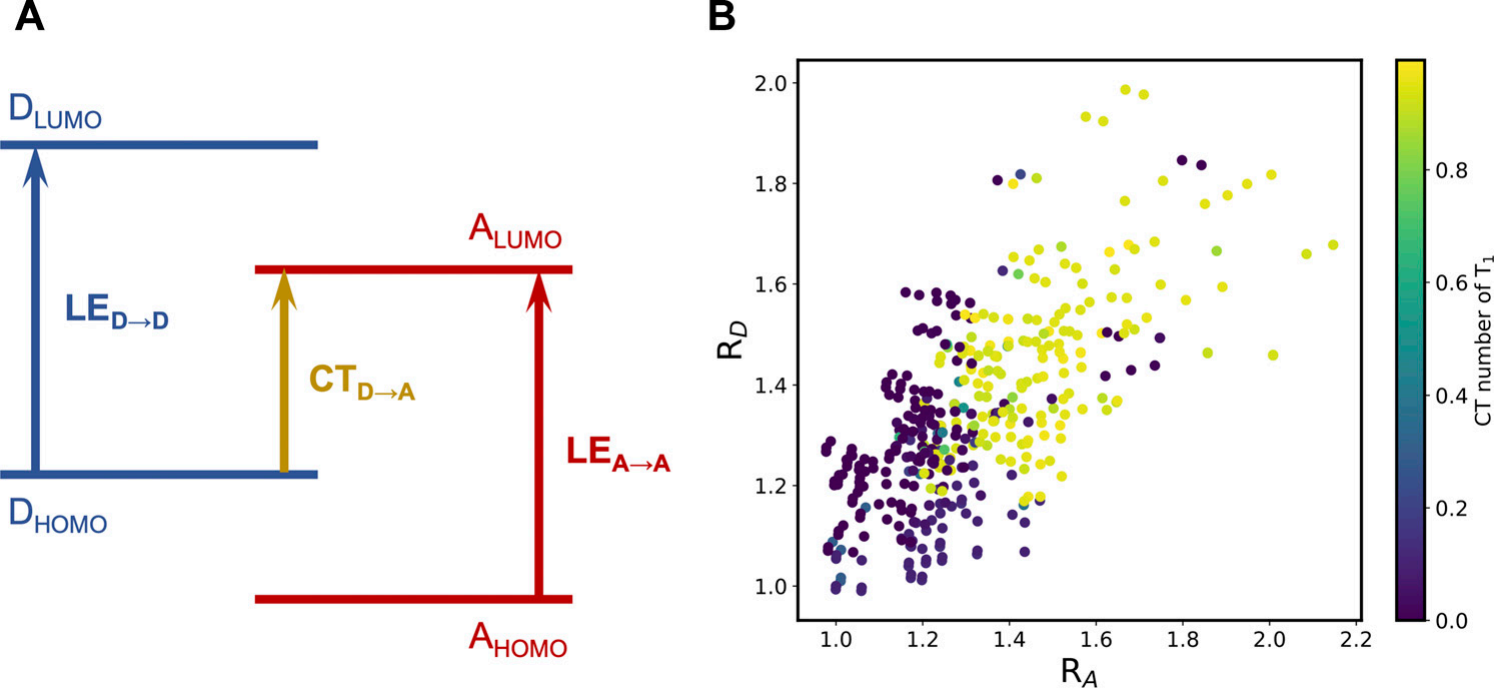
**(A)** Schematic representation of the relation between the competence of LE and CT states and the relative order of FMO energies; **(B)** R_D_-R_A_ scatter plots colored by the CT number of the T_1_ state of the A- π -D- π -A database (433 molecules).

The emergence of the LE states can be explained utilizing the frontier molecular orbital (FMO) energies of the constituent building blocks (Blaskovits et al., 2020), which is illustrated in Figure 6A. The competition between the CT excitation and LE excitation depends on the relative ordering of the FMOs. In this context, we can define two descriptors, $R_{A}=\left(E_{\text{LUMO}}^{A}-E_{\text{HOMO}}^{A}\right)/\left(E_{\text{LUMO}}^{A}-E_{\text{HOMO}}^{D}\right)$ , $R_{D}=\left(E_{\text{LUMO}}^{D}-E_{\text{HOMO}}^{D}\right)/\left(E_{\text{LUMO}}^{A}-E_{\text{HOMO}}^{D}\right)$ , where $E_{\text{LUMO}/\text{HOMO}}^{A/D}$ are the LUMO/HOMO energies of the acceptor/donor. If the $R_{A}$ or the $R_{D}$ is much larger than 1, the CT excitation is more favorable than the LE for the low-lying excited states and vice versa. Figure 6B demonstrates that this simple approximation works quite well for our A- π -D- π -A database: For $R_{A}$ or $R_{D}$ smaller than ∼1.2, the CT number of T_1_ becomes close to 0. The same behavior was also found in the D- π -A- π -D database, as shown in Supplementary Figure S5. This indicates that a prescreening step based on the individual building blocks saves the computational cost, similar to pre-screening of singlet fission donor-acceptor copolymers (Blaskovits et al., 2020).

### Compounds With T_n_ States Lying Close to S_1_

The S_1_ and T_1_ states of most molecules that pass the first screening step ( $\text{Δ}E_{\text{ST}}$ < 0.1 eV) exhibit CT character. According to the El-Sayed rule, the $k_{\text{rISC}}$ is zero between two states having the same excited-state character, which implies that the rISC may not occur for these pre-screened compounds. However, the conformational disorder present in the solid state leads to a distribution of dihedral angles between the constituent donor and acceptor (Weissenseel et al., 2019). This disorder gives rise to different excited-state characters, that is different mixing of CT and LE diabatic states of the S_1_ and T_1_ states, (de Silva et al., 2019), resulting in non-zero $k_{\text{rISC}}$ . This explains why TADF could still be observed in the thin film of CzDBA, where CT_S1_ and CT_T1_ are both close to 1 in the gas phase (Kotadiya et al., 2019a).

In addition, higher triplet states (T_*n*_ with n>1 ) with different excited-state character from that of S_1_, can also assist in the rISC process *via* a two-step mechanism (Gibson et al., 2016). A large second order coupling can be achieved when the energies of S_1_, T_1_ and T_*n*_ are close to each other. Compounds with close-lying S_1_ and T_1_ that already show different excited-state characters would possess large first-order coupling and hence high *k*_rISC_. Therefore, we applied additional screening criteria to the as-screened ∼500 molecules: 1) there should be at least one triplet state T_n_ that is close to S_1_ (| E_S_1__− E_T*_n_*_ | < 0.1 eV); 2) for the triplet states that are energetically close to S_1_, the difference between the CT numbers of S_1_ and T_n_ should be larger than 0.5 (CT_S_1__−CT_T*_n_*_ > 0.5) to give reasonable spin-orbit coupling.

**FIGURE 7:**
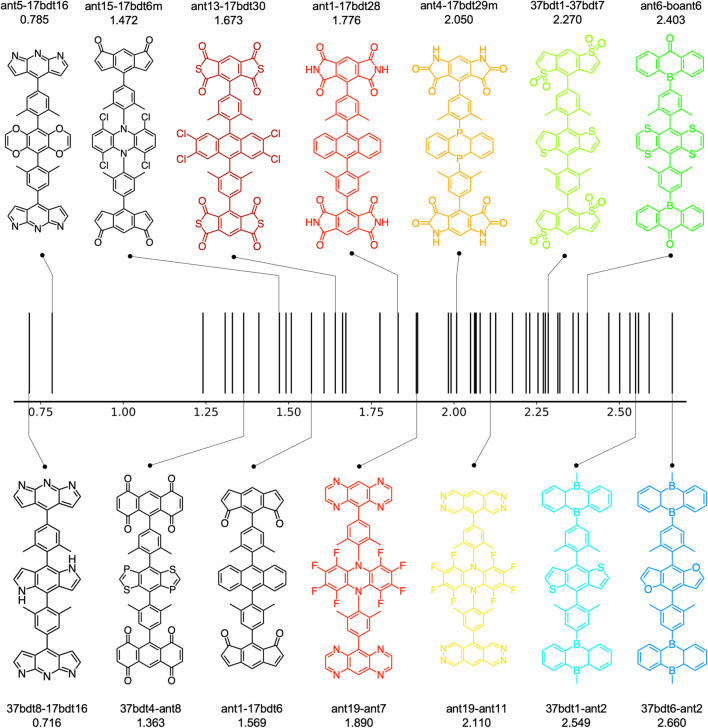
The estimated EL spectrum maximum of 49 A- π -D- π -A candidates of single-layer OLED emitters. The molecular structures of the 14 selected compounds are depicted.

Overall, around 100 molecules pass the criteria (49 A- π -D- π -A and 46 D- π -A- π -D), where the molecular structures are summarized in Figure 7. All of these compounds, except for A- π -D- π -A molecules with non-centrosymmetric core boant4 ( D=4−5 Debye), possess nearly zero molecular dipole moment. Therefore, they are considered promising candidates for single-layer OLED emitters. The position of the electroluminescence (EL) spectrum maximum of each compound, as shown in Figure 7, was estimated by subtracting the computed S_1_ energy by a value δ, which is defined as $\delta =E_{S_{1}}-\lambda_{EL,max}=2.480-2.214=0.266\;\text{eV}$ , where E_S_1__ and *λ*_EL,max_ is the experimental optical gap and the wavelength of the EL spectrum maximum of CzDBA (Kotadiya et al., 2019a). These values are listed in Supplementary Tables S1,S2. We obtained a series of potential TADF emitters with various EL spectrum maximum, ranging from infrared (0.716 eV) to blue color (2.660 eV), which paves the way for future development of single-layer OLED devices.

### Charge Carrier Density of States

**FIGURE 8:**
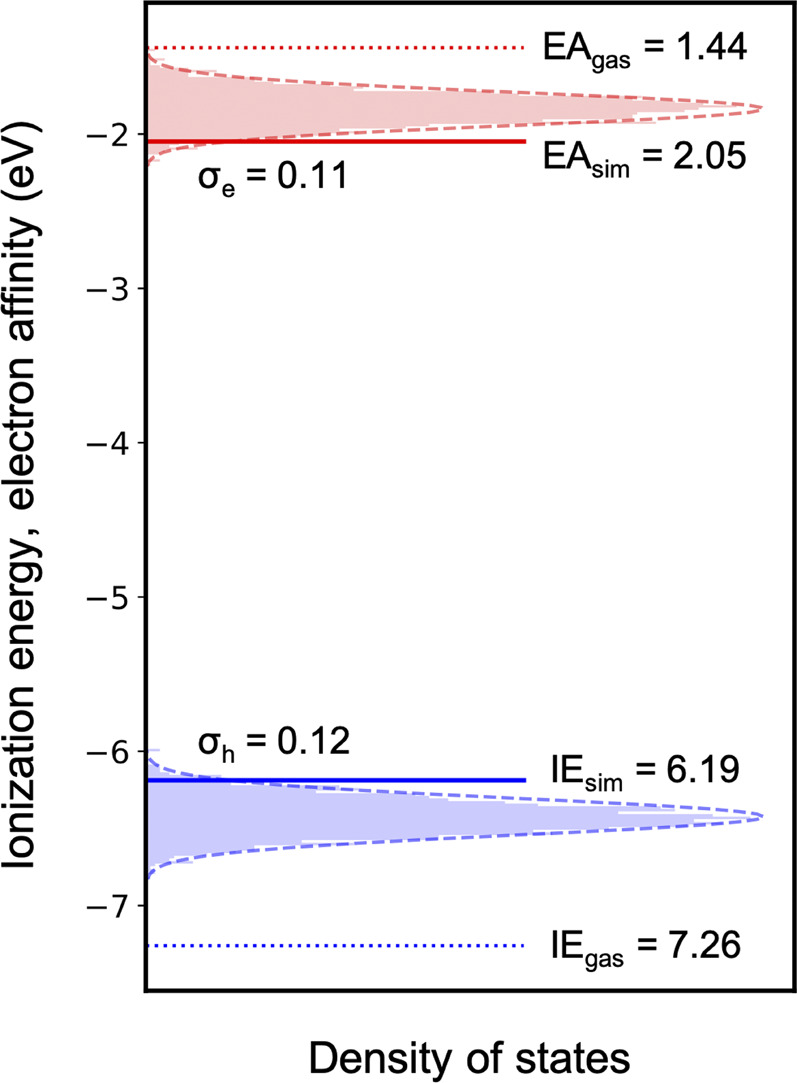
Simulated ionization energy and electron affinity distribution in an amorphous 37bdt1-ant2 film.

More sophisticated solid-state simulations can then be performed for the much smaller molecular dataset, which is now only ∼10% of the initial number of compounds. As a proof of concept, we computed the charge carrier density of states for the blue A- π -D- π -A emitter, 37bdt1-ant2 (as shown in Figure 8). The amorphous simulated morphology was generated using molecular dynamics, where the details can be found in Supplementary Note S4. The energetic disorder for electrons (0.11 eV) and holes (0.12 eV) is relatively small, which indicates a good hole/electron mobility. This also demonstrates the success of our design strategy regarding small molecular dipole moment. Since the simulated IE and EA of 37bdt1-ant2 lie at the border of the trap-free window, further experimental measurements are necessary to verify if it is really free from universal traps.

## Conclusion

To summarize, we have provided clear design rules for single-layer OLED materials comprising TADF:

1. Molecular gas-phase ionization energies and electron affinities within the ∼ 6.2 eV to ∼ 2.0 eV range. These are calculated using implicit solvent with the dielectric constant of 3 and ensure trap-free transport of electrons and holes.
2. Small molecular dipole moment. This condition is imposed by the molecular symmetry and ensures a narrow density-of-states distribution in the solid state.
3. Small singlet-triplet splitting. This is provided by the orthogonal alignment of the bridge and the core units, as well as the suitable level alignment between the HOMO and LUMO of the donor and acceptor units. This is required for efficient reverse intersystem crossing.
4. Different character of singlet and triplet excitations to ensure sufficient spin-orbit coupling that enables reverse intersystem crossing.

Using the suggested design rules, we have proposed a set of TADF emitters with a broad range of emission wavelengths, from infrared to sky-blue. We hope that the suggested structures can serve as a clear guide towards further development of efficient and stable single-layer OLEDs.

## Data Availability

The datasets presented in this study can be found in online repository: https://gitlab.mpcdf.mpg.de/materials/tadf-screening.
