# Supplementary material for: Virtual Screening of TADF Emitters for Single-Layer OLEDs
Source: Front Chem. 2021 Dec 16;9:800027. doi: 10.3389/fchem.2021.800027 (PMC8716429; doi:10.3389/fchem.2021.800027)
Supplement: Supplementary file 1 [file DataSheet1.PDF]

Supplementary information

Kun-Han Lin[a,*] , Gert-Jan A. H. Wetzelaer, Paul W. M. Blom, Denis Andrienko[b,*]

[1] Max Planck Institute for Polymer Research, Ackermannweg 10, 55128 Mainz, Germany

*Corresponding authors

[a]Email: link@mpip-mainz.mpg.de

[b]Email: denis.andrienko@mpip-mainz.mpg.de

Supplementary Note 1

The molecular structures of the initial 97 building blocks are shown in **Figure S1**. As stated in the main text, we only choose the molecular fragments that fulfill the following criteria: they are (1) (quasi-)linear, (2) composed of three (fused) rings and (3) are reported in literature (synthesizable). We did not divide them into acceptor and donor subcategories at the beginning. Instead, we classified them based on our computed gas-phase ionization energy (IE) and electron affinity (EA), as shown in **Figure S2**.

The geometry optimization of the 97 building blocks was performed using DFT at ωB97X-D/def2-TZVP level of theory and Gaussian16 (Frisch et al., 2016). The prescreening criteria (gas-phase IE and EA) were then computed using an omega-tuning procedure LC-ωPBE*/may-cc-pVTZ. We then construct a database $\mathbf{DB_{blcoks}}$ for these “trap-free” building blocks, which contains the information, including gas-phase IE and EA, optimized geometry, optimal ω value, and SMILES.

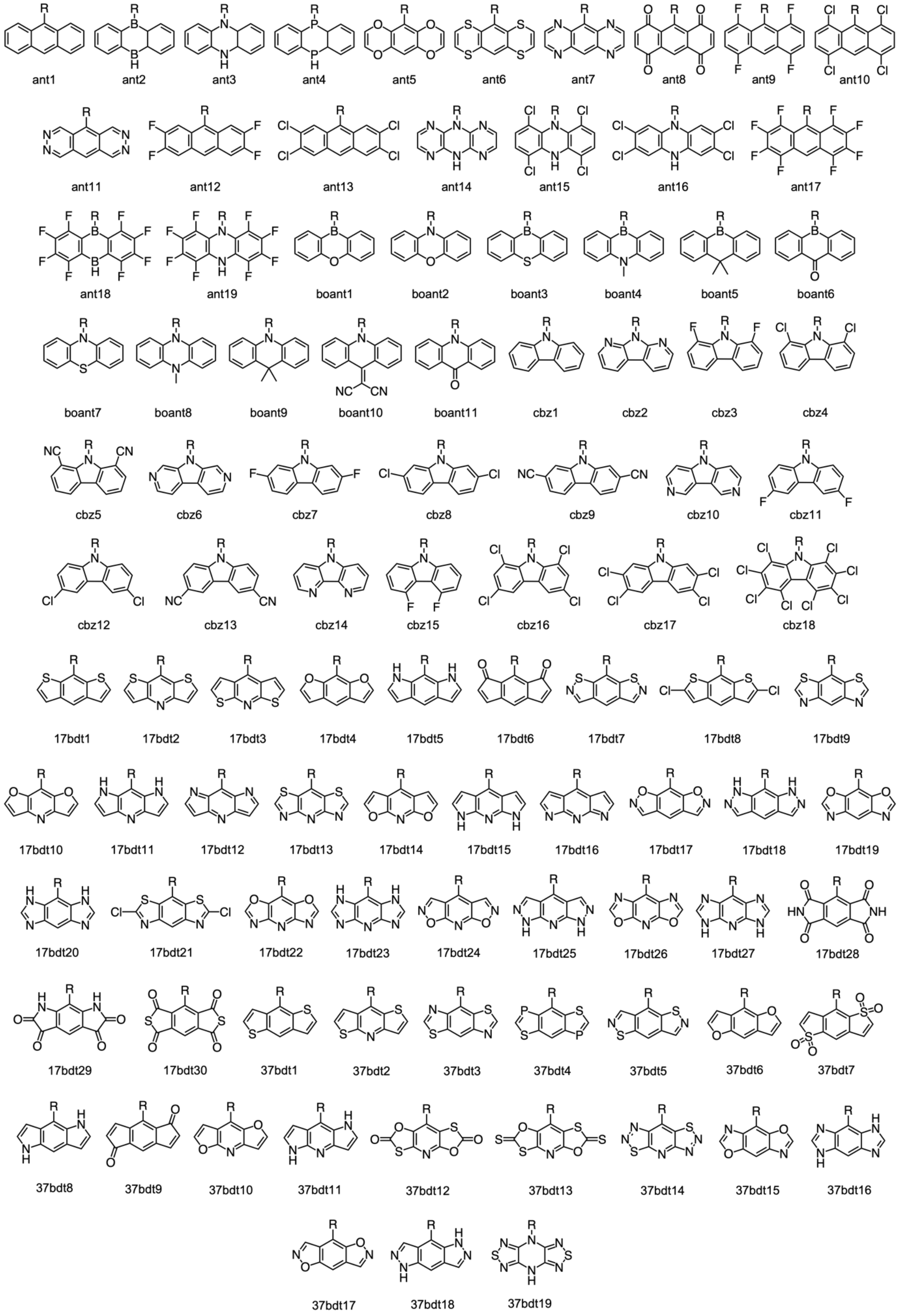

Figure S1. Molecular structures and names of the initial 97 building blocks.

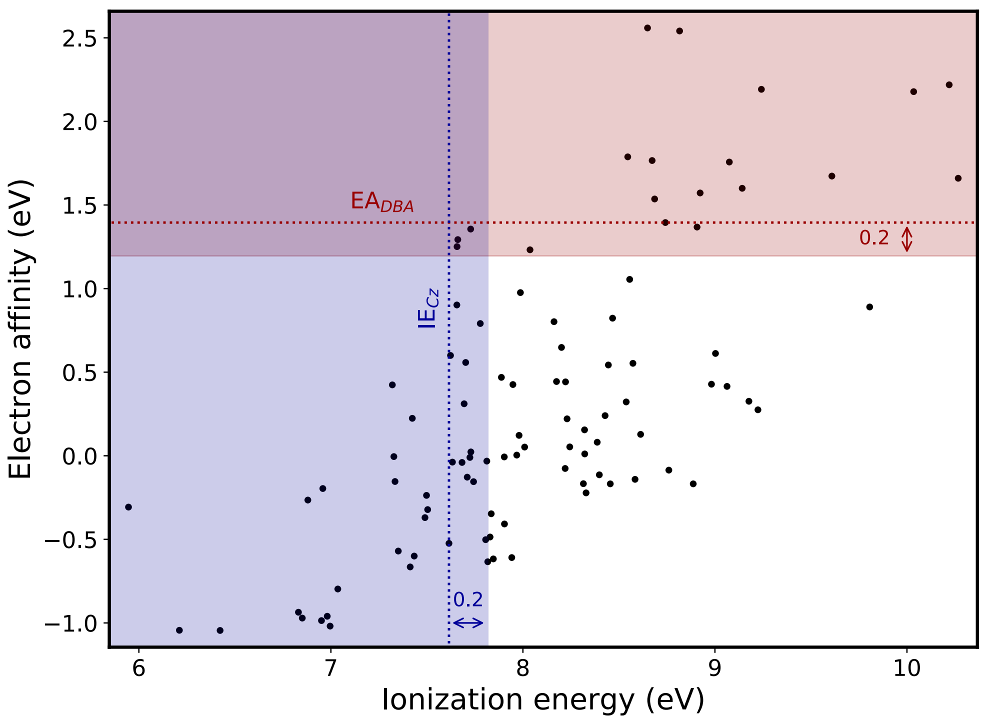

Figure S2. Gas-phase ionization energy and electron affinity of 97 molecular building blocks investigated in this work. The blue and the red dotted lines represent the IE and EA of carbazole (Cz) and 5,10-dihydroboranthrene (DBA), respectively. A 0.2 eV window is applied to include more donors and acceptors into the database.

Supplementary Note 2

All DFT and TD-DFT computations were performed using Gaussian16.

Construction and optimization of molecular geometry

Since the compounds are constructed from the core and the arm building blocks, their names are given as $Name_{core}$-$Name_{arm}$. For example, the name of CzDBA using this nomenclature is ant2-cbz1. If there is an “m” character at the end of $Name_{arm}$, it means that the substitution site is at the *para* position to the one (-R) shown in **Figure 2**. The initial 3D molecular geometry of each compound was constructed from the SMILES string, which are generated

from individual building blocks as shown in **Scheme S1**, using gen3d operation with GAFF force field (Wang et al., 2004) implemented in OpenBabel (O'Boyle et al., 2011). This was followed by two subsequent optimization steps to speed up the overall geometry optimization: first optimization with a semi-empirical (GFN2-xTB) method (Bannwarth et al., 2019) and then with DFT (ωB97X-D/def2-SVP).

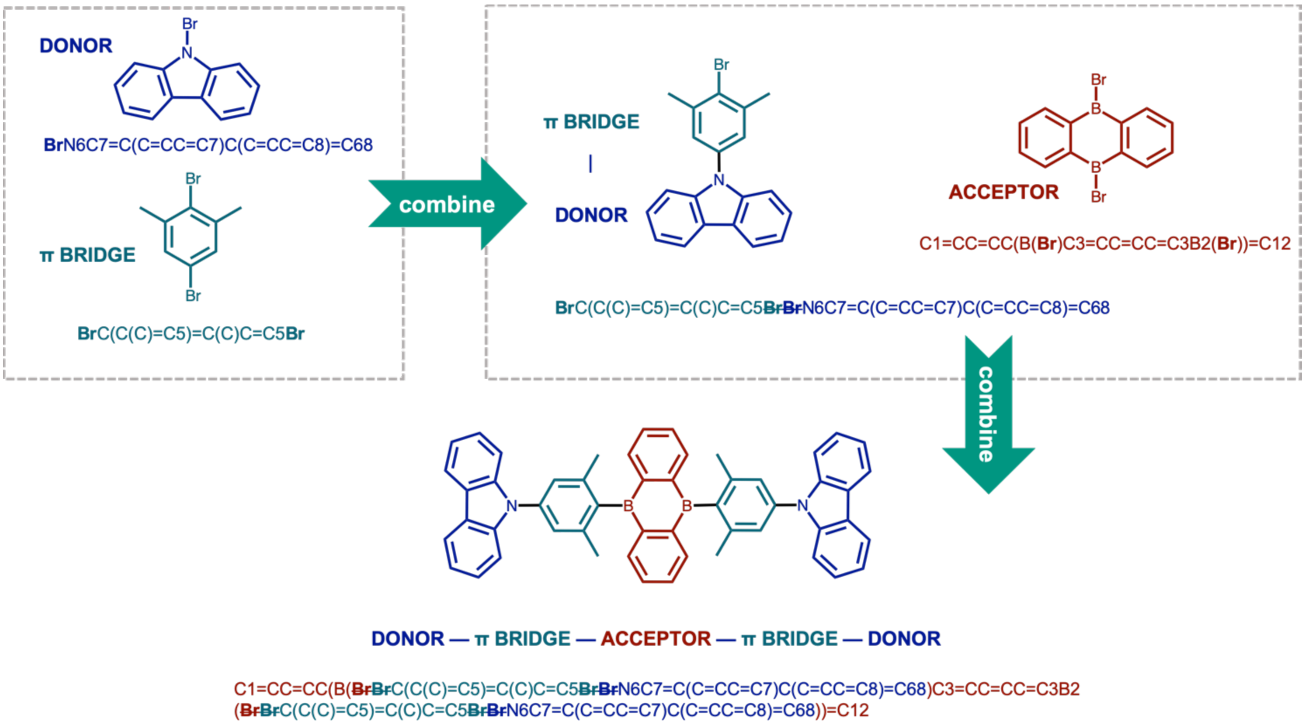

Scheme S1. Schematic workflow of constructing SMILES of D-π-A-π-D compound form individual building blocks.

ω-tuning procedure

To obtain reliable predictions of solid-state IE, EA and excited-state energy, we followed the cost-effective ω-tuning protocol with PCM implicit solvent model (ε = 3.0) (Sun et al., 2016, 2017). The optimal ω can be obtained by introducing the target function (J) to be minimized, defined as

$$J(\omega) = \left|IE_N(\omega) + \epsilon_{N,HOMO}(\omega)\right| + \left|IE_A(\omega) + \epsilon_{A,HOMO}(\omega)\right|$$

where the capital N and A stands for the neutral and anionic states, respectively. The computations were performed at ωB97X-D/def2-TZVP level of theory. The optimal ω for each compound was stored in the database, ranging from 0.010 to 0.040 Bohr^{-1}. This ω range is close to the values reported in the literatures (Sun et al., 2016, 2017).

Excited-state computations

For the description of excited states, we employ linear-response time-dependent density functional theory with Tamm-Dancoff approximation (TDA/TD-DFT) along with ωB97X-D*/def2-TZVP (with the optimal ω) to compute the excitation energies and oscillator strengths.

Excited-state character analysis

The excited-state characters of singlet and triplet states were analyzed using fragment-based analysis method implemented in TheoDORE (Plasser, 2020). As stated in the main text, we divide each molecule into two fragments: (1) the core fragment (f_C) and the rest (two bridge+arm pairs, f_A). We used the Löwdin style analysis (Om_formula=2) to calculated the CT number, where CT = 1 represents a 100% CT character while CT = 0 shows a local-excitation (LE) character.

Supplementary Note 3

Although the discussions in the main text are mainly based on the A-π-D-π-A compounds, we found very similar behavior in D-π-A-π-D compounds as well. Therefore, we put the results of D-π-A-π-D compounds, **Figure S3-S5**, which are parallel to **Figure 4-6** in this note.

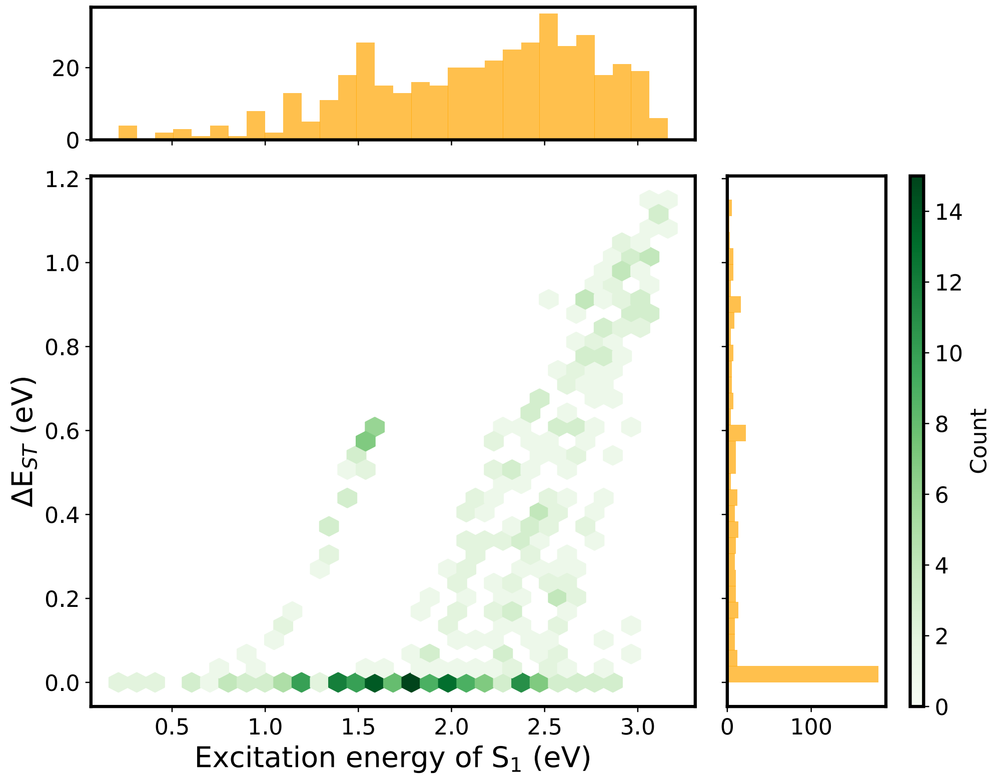

Figure S3. 2D histogram constructed using the descriptors (E_{S1}, ΔE_{ST}) of the D-π-A-π-D database (481 molecules). The corresponding 1D histogram for each descriptor is shown on the axes.

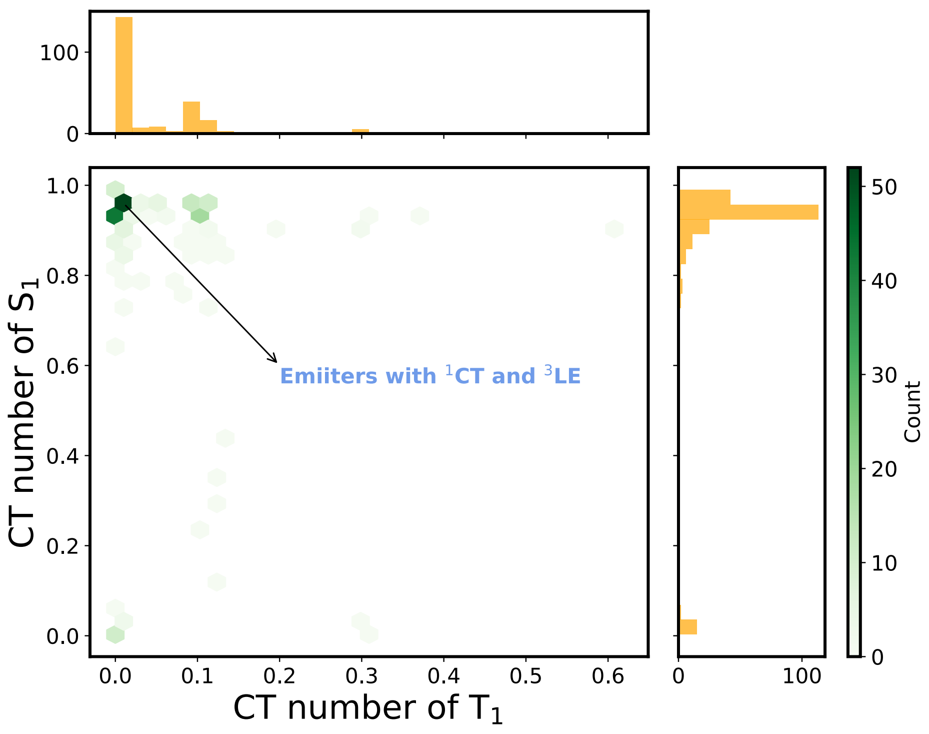

Figure S4. 2D histogram constructed using the CT numbers of T_1 and S1 states of the D-π-A-π-D molecules with ΔE_{ST} > 0.1 eV (213 molecules). The corresponding 1D histogram for each descriptor is shown on the axes.

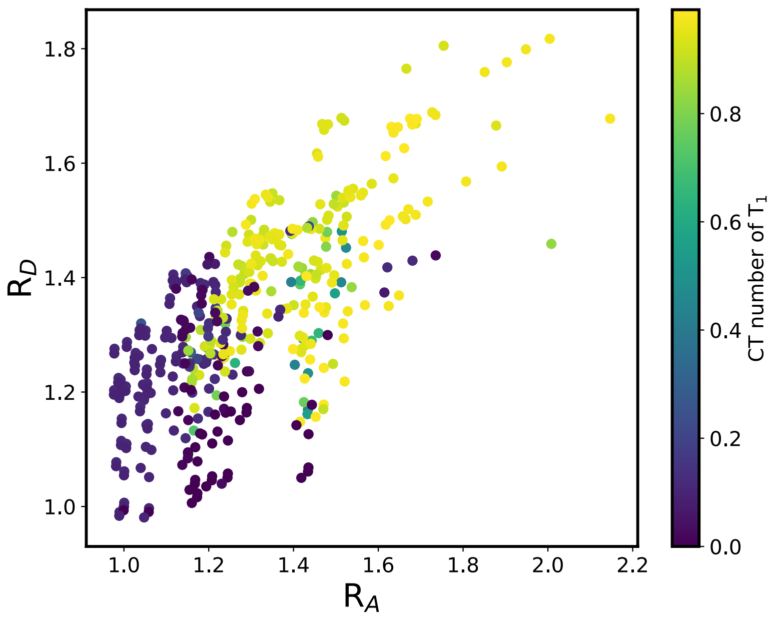

Figure S5. R_D-R_A scatter plots colored by the CT number of the T_1 state of the D-π-A-π-D database (481 molecules).

Supplementary Note 4

Table S1. The estimated EL spectrum maximum of 49 A-π-D-π-A candidates of single-layer OLED emitters.

Name	EL_{max} (eV)	Name	EL_{max} (eV)
37bdt8-17bdt16	0.716	ant14-ant11	2.065
ant5-17bdt16	0.785	ant14-17bdt29	2.067
boant4-ant8	1.241	37bdt1-17bdt29m	2.079
ant9-ant8	1.308	ant19-ant11	2.11
ant10-ant8	1.33	ant19-17bdt29	2.126
37bdt4-ant8	1.364	boant4-37bdt14	2.177
ant13-ant8	1.41	37bdt18-37bdt7	2.218
ant15-17bdt6m	1.472	37bdt19-37bdt7	2.229
ant5-17bdt6m	1.492	ant14-ant2	2.254
ant19-17bdt6	1.508	37bdt1-37bdt7	2.27
ant1-17bdt6	1.569	ant4-37bdt7	2.276
ant10-17bdt30	1.607	37bdt19-boant6	2.285
37bdt18-17bdt6	1.641	ant6-37bdt7	2.314
37bdt19-17bdt6	1.663	37bdt6-37bdt14	2.32
ant13-17bdt30	1.673	37bdt1-boant6	2.36
ant1-17bdt28	1.776	boant4-boant6	2.376
ant16-ant10	1.776	ant6-boant6	2.403
ant14-ant7	1.831	37bdt19-ant2	2.468
ant16-ant17	1.886	37bdt1-ant18	2.501
ant19-ant7	1.89	37bdt6-boant6	2.532
ant15-ant2	1.983	37bdt1-ant2	2.549
37bdt19-17bdt29m	1.991	boant4-ant2	2.558
37bdt18-17bdt29m	2.008	ant6-ant2	2.59
ant4-17bdt29m	2.05	37bdt6-ant2	2.66
37bdt4-17bdt28	2.062		

Table S2. The estimated EL spectrum maximum of 46 D-π-A-π-D candidates of single-layer OLED emitters.

Name	EL_{max} (eV)	Name	EL_{max} (eV)
ant8-boant11	1.364	17bdt28-ant12	1.988
ant8-ant6	1.365	ant11-17bdt15	2.022
ant8-boant10	1.406	ant11-17bdt18	2.049
37bdt7-ant14	1.451	ant11-37bdt18	2.06
37bdt9-ant14	1.463	ant11-ant19	2.097
ant8-ant12	1.474	ant11-cbz11	2.118
37bdt9-37bdt11	1.479	ant2-37bdt18	2.129
37bdt9-17bdt11	1.485	ant11-37bdt19	2.131
17bdt30-ant10	1.656	ant11-cbz1	2.144
17bdt28-ant1	1.754	37bdt7-17bdt1	2.145
ant17-boant2	1.781	ant11-17bdt1m	2.165
17bdt28-37bdt11	1.788	ant11-17bdt18m	2.169
ant18-ant1	1.793	ant11-37bdt1	2.17
ant7-17bdt18	1.836	37bdt7-cbz12	2.189
ant7-37bdt18	1.85	ant11-17bdt1	2.195
ant18-ant12	1.926	ant2-17bdt18m	2.229
ant7-cbz11	1.934	37bdt7-ant6	2.235
ant18-boant10	1.944	37bdt7-37bdt6	2.244
17bdt28-boant10	1.952	ant2-37bdt19	2.296
ant7-cbz1	1.953	37bdt7-17bdt8m	2.317
ant7-17bdt18m	1.956	ant2-boant4	2.512
ant7-37bdt19	1.963	ant2-boant3	2.634
ant7-ant19	1.966	ant2-ant4	2.635

Supplementary Note 5

Our protocol for generating amorphous morphology of 37bdt1-ant2 involves two steps: (i) forcefield parameterization and (ii) classical molecular dynamics (MD) simulations. The simulated amorphous morphology was used for charge-carrier DOS computations. The details are described as following.

Forcefield parameterization

All bonded parameters apart from proper and improper dihedrals were taken from OPLS-AA force field(Jorgensen et al., 1996; Jorgensen and Tirado-Rives, 2005) and our previous work (Mondal et al., 2021). The non-bonded parameters, atomic partial charges and Lennard-Jones parameters, were derived following the protocol proposed by Cole et al.(Cole et al., 2016). In short, the overlapping atomic electron densities were obtained via the density-derived electrostatic and chemical (DDEC6) electron density partitioning scheme (Manz and Limas, 2016). The atomic partial charges can then be obtained by integrating the corresponding atomic electron densities over the whole space. Additionally, the two parameters, A and B, in Lennard-Jones potential are then derived using Tkatchenko−Scheffler (TS) scheme (Tkatchenko and Scheffler, 2009), where the radius of the free atom in a vacuum ($\mathrm{R_i^{free}}$) is taken from ref. (Cole et al., 2016). The electron density was obtained using Gaussian16 at ωB97X-D/6-311G(d,p) level and the DDEC6 computations were performed using Chargemol of version 09_26_2017 (Manz and Limas, 2016). The molecule was partitioned into several rigid fragments, following the same procedure as our previous work (Mondal et al., 2021). After nonbonded parameters were set, the dihedral potentials that connect these rigid fragments, which are usually missing in the OPLS-AA database, were parameterized using the constrained optimization scanning performed at ωB97X-D/6-311G(d,p) level using Gaussian16. The reason for using 6-

311G(d,p) basis set instead of def2-TZVP is due to the much lower cost of the former, especially in optimization steps. Yet, the relative potential energies from the dihedral scanning obtained using these two basis sets are close to each other. For more details of the parameterization of dihedral potentials, please refer to ref. (Poelking et al., 2013).

Classical MD simulations

All classical MD simulations were performed using GROMACS version 2020.3 (Pronk et al., 2013; Abraham et al., 2015). For the long-range electrostatic interactions, the particle mesh Ewald (PME) method was employed with a 0.12 nm Fourier spacing. A cutoff of 13 Å was applied to all non-bonded interactions. The temperature and pressure control were accomplished using velocity rescaling with a stochastic term (Bussi et al., 2007) (τ_T = 0.5 ps) and an isotropic coupling for the pressure from a Berendsen barostat (P_0 = 1 bar, $\chi = 4.5 \times 10^{-5}$ bar^{-1} , and τ_P = 0.5 ps).

For 37bdt1-ant2, 2000 molecules were initially randomly placed in a simulation box with a low target density around 50 to 150 kg m^{-3} using Packmol (Martínez et al., 2009). The whole system was then heated from 100K to 300K at a rate of 0.67 K ps^{-1}. It was then equilibrated at 300K until the density reached a steady value. This step helps to prevent the system from exploding due to a high heating rate. Finally, the system was heated from 300K to 800K at a rate of 0.5 K ps^{-1}, followed by an equilibration at 800K for 10ns. The equilibration time is long enough to ensure a steady density of the system for all compounds discussed here. Finally, the system underwent a linear cooling procedure from 800K to 300K at a 100K ns^{-1} cooling rate and then was equilibrated at 300K for 10 ns. The last snapshot of the trajectory was used for further charge carrier DOS computations.

Charge carrier DOS computations

The ionization energy (IE) and electron affinity (EA) in the bulk were computed using a perturbative way:

$$O_{bulk} = O_{gas} + \Delta O_{solid}$$

where the O_{bulk} is the target bulk property, O_{gas} is the target gas-phase property computed using density-functional theory (DFT) and ΔO_{solid} is the solid-state stabilization computed using the Thole model. The computational details of atomistic simulations are described below.

Gas-phase computations

All the molecular geometries were optimized using DFT at ωB97X-D/6-311G(d,p) level of theory. The vertical ionization energy (IE) and electron affinity (EA) were evaluated using a ΔSCF method:

$$IE_{gas} = E_{gas}^{cN} - E_{gas}^{nN}$$

$$EA_{gas} = E_{gas}^{nN} - E_{gas}^{aN}$$

where $E_{gas}^{nN} / E_{gas}^{cN} / E_{gas}^{aN}$ is the total energy of the neutral/cationic/anionic (in lower case) molecule in neutral ground-state geometry (in uppercase). All gas-phase computations were performed using Gaussian16.

Atomic multipoles and atomic polarizabilities

The atomic multipoles for all states (nN, cN and aN) were derived using the GDMA program (Stone, 2005). The input (density matrix) for GDMA is coming from the gas-phase computations described above.

The atomic polarizabilities of neutral and charged ground state (nN, cN and aN) were obtained by linearly scaling the isotropic atomic polarizabilities of AMOEBA force field (Ren and Ponder, 2002). The scaling factor is the value when the effective molecular polarizable volumes of the QM computations and this method match.

Solid-state stabilization

The computational protocol of solid-state stabilization using the VOTCA-CTP package is described in our previous work (Rühle et al., 2011; Poelking and Andrienko, 2016). Overall, the QM-derived atomic multipoles and atomic polarizabilities are mapped onto each site (atom) of the morphology. The electrostatic and induction contributions to the site energies were calculated self-consistently using the Thole model. This approach, in combination with an aperiodic inclusion of charges and excitons to a neutral periodic morphology, is implemented in VOTCA-CTP.

References

Abraham, M. J., Murtola, T., Schulz, R., Páll, S., Smith, J. C., Hess, B., et al. (2015). GROMACS: High performance molecular simulations through multi-level parallelism from laptops to supercomputers. *SoftwareX* 1–2, 19–25. doi:10.1016/j.softx.2015.06.001.

Bannwarth, C., Ehlert, S., and Grimme, S. (2019). GFN2-xTB—An Accurate and Broadly Parametrized Self-Consistent Tight-Binding Quantum Chemical Method with Multipole Electrostatics and Density-Dependent Dispersion Contributions. *J. Chem. Theory Comput.* 15, 1652–1671. doi:10.1021/acs.jctc.8b01176.

Bussi, G., Donadio, D., and Parrinello, M. (2007). Canonical sampling through velocity rescaling. *J. Chem. Phys.* 126. doi:10.1063/1.2408420.

Cole, D. J., Vilseck, J. Z., Tirado-Rives, J., Payne, M. C., and Jorgensen, W. L. (2016).

Biomolecular Force Field Parameterization via Atoms-in-Molecule Electron Density Partitioning. *J. Chem. Theory Comput.* 12, 2312–2323. doi:10.1021/acs.jctc.6b00027.

Frisch, M. J., Trucks, G. W., Schlegel, H. B., Scuseria, G. E., Robb, M. A., Cheeseman, J. R., et al. (2016). Gaussian 16, Revision B.01. Gaussian, Inc., Wallingford CT.

Jorgensen, W. L., Maxwell, D. S., and Tirado-Rives, J. (1996). Development and Testing of the OPLS All-Atom Force Field on Conformational Energetics and Properties of Organic Liquids. *J. Am. Chem. Soc.* 118, 11225–11236. doi:10.1021/ja9621760.

Jorgensen, W. L., and Tirado-Rives, J. (2005). Potential energy functions for atomic-level simulations of water and organic and biomolecular systems. *Proc. Natl. Acad. Sci.* 102, 6665–6670. doi:10.1073/pnas.0408037102.

Manz, T. A., and Limas, N. G. (2016). Introducing DDEC6 atomic population analysis: part 1. Charge partitioning theory and methodology. *RSC Adv.* 6, 47771–47801. doi:10.1039/C6RA04656H.

Martínez, L., Andrade, R., Birgin, E. G., and Martínez, J. M. (2009). PACKMOL: A package for building initial configurations for molecular dynamics simulations. *J. Comput. Chem.* 30, 2157–2164. doi:10.1002/jcc.21224.

Mondal, A., Paterson, L., Cho, J., Lin, K.-H., van der Zee, B., Wetzelaer, G.-J. A. H., et al. (2021). Molecular library of OLED host materials—Evaluating the multiscale simulation workflow. *Chem. Phys. Rev.* 2, 031304. doi:10.1063/5.0049513.

O’Boyle, N. M., Banck, M., James, C. A., Morley, C., Vandermeersch, T., and Hutchison, G. R. (2011). Open Babel: An open chemical toolbox. *J. Cheminform.* 3, 33. doi:10.1186/1758-2946-3-33.

Plasser, F. (2020). TheoDORE: A toolbox for a detailed and automated analysis of electronic excited state computations. *J. Chem. Phys.* 152, 084108. doi:10.1063/1.5143076.

Poelking, C., and Andrienko, D. (2016). Long-Range Embedding of Molecular Ions and Excitations in a Polarizable Molecular Environment. *J. Chem. Theory Comput.* 12, 4516–4523. doi:10.1021/acs.jctc.6b00599.

Poelking, C., Cho, E., Malafeev, A., Ivanov, V., Kremer, K., Risko, C., et al. (2013). Characterization of Charge-Carrier Transport in Semicrystalline Polymers: Electronic Couplings, Site Energies, and Charge-Carrier Dynamics in Poly(bithiophene- alt -thienothiophene) [PBTTT]. *J. Phys. Chem. C* 117, 1633–1640. doi:10.1021/jp311160y.

Pronk, S., Páll, S., Schulz, R., Larsson, P., Bjelkmar, P., Apostolov, R., et al. (2013). GROMACS 4.5: a high-throughput and highly parallel open source molecular simulation toolkit. *Bioinformatics* 29, 845–854. doi:10.1093/bioinformatics/btt055.

Ren, P., and Ponder, J. W. (2002). Consistent treatment of inter- and intramolecular polarization in molecular mechanics calculations. *J. Comput. Chem.* 23, 1497–1506. doi:10.1002/jcc.10127.

Rühle, V., Lukyanov, A., May, F., Schrader, M., Vehoff, T., Kirkpatrick, J., et al. (2011). Microscopic simulations of charge transport in disordered organic semiconductors. *J. Chem. Theory Comput.* 7, 3335–3345. doi:10.1021/ct200388s.

Stone, A. J. (2005). Distributed Multipole Analysis : Stability for Large Basis Sets Distributed Multipole Analysis : Stability for Large Basis Sets. *Analysis* 1, 1128–1132. doi:10.1021/ct050190+.

Sun, H., Hu, Z., Zhong, C., Chen, X., Sun, Z., and Brédas, J. L. (2017). Impact of Dielectric Constant on the Singlet-Triplet Gap in Thermally Activated Delayed Fluorescence Materials. *J. Phys. Chem. Lett.* 8, 2393–2398. doi:10.1021/acs.jpclett.7b00688.

Sun, H., Ryno, S., Zhong, C., Ravva, M. K., Sun, Z., Körzdörfer, T., et al. (2016). Ionization Energies, Electron Affinities, and Polarization Energies of Organic Molecular Crystals: Quantitative Estimations from a Polarizable Continuum Model (PCM)-Tuned Range-

Separated Density Functional Approach. *J. Chem. Theory Comput.* 12, 2906–2916. doi:10.1021/acs.jctc.6b00225.

Tkatchenko, A., and Scheffler, M. (2009). Accurate Molecular Van Der Waals Interactions from Ground-State Electron Density and Free-Atom Reference Data. *Phys. Rev. Lett.* 102, 073005. doi:10.1103/PhysRevLett.102.073005.

Wang, J., Wolf, R. M., Caldwell, J. W., Kollman, P. A., and Case, D. A. (2004). Development and testing of a general amber force field. *J. Comput. Chem.* 25, 1157–1174. doi:10.1002/jcc.20035.
